# Fluid–Solid Interaction Analysis for Developing In-Situ Strain and Flow Sensors for Prosthetic Valve Monitoring

**DOI:** 10.3390/s24155040

**Published:** 2024-08-04

**Authors:** Silvia Puleo, Salvatore Pasta, Francesco Scardulla, Leonardo D’Acquisto

**Affiliations:** 1 Department of Engineering, University of Palermo, 90128 Palermo, Italy; silvia.puleo01@unipa.it (S.P.); salvatore.pasta@unipa.it (S.P.); francesco.scardulla@unipa.it (F.S.); 2 Department of Research, Scientific Institute of Hospitalization and Care-Mediterranean Institute for Transplantation and Highly Specialized Therapies (IRCCS-ISMETT), Via Tricomi, 5, 90127 Palermo, Italy

**Keywords:** transcatheter aortic valve implantation (TAVI), fluid–structure interaction (FSI), photoplethysmography (PPG) sensor

## Abstract

Transcatheter aortic valve implantation (TAVI) was initially developed for adult patients, but there is a growing interest to expand this procedure to younger individuals with longer life expectancies. However, the gradual degradation of biological valve leaflets in transcatheter heart valves (THV) presents significant challenges for this extension. This study aimed to establish a multiphysics computational framework to analyze structural and flow measurements of TAVI and evaluate the integration of optical fiber and photoplethysmography (PPG) sensors for monitoring valve function. A two-way fluid–solid interaction (FSI) analysis was performed on an idealized aortic vessel before and after the virtual deployment of the SAPIEN 3 Ultra (S3) THV. Subsequently, an analytical analysis was conducted to estimate the PPG signal using computational flow predictions and to analyze the effect of different pressure gradients and distances between PPG sensors. Circumferential strain estimates from the embedded optical fiber in the FSI model were highest in the sinus of Valsalva; however, the optimal fiber positioning was found to be distal to the sino-tubular junction to minimize bending effects. The findings also demonstrated that positioning PPG sensors both upstream and downstream of the bioprosthesis can be used to effectively assess the pressure gradient across the valve. We concluded that computational modeling allows sensor design to quantify vessel wall strain and pressure gradients across valve leaflets, with the ultimate goal of developing low-cost monitoring systems for detecting valve deterioration.

## 1. Introduction

Transcatheter aortic valve implantation (TAVI) has become the standard of care for elderly patients with aortic stenosis, as compared to conventional open-chest surgery [1]. Ongoing clinical trials are currently investigating the feasibility and safety of TAVI in young and bicuspid patients, who were previously excluded from previous clinical trials [2,3,4,5]. However, the long-term durability of transcatheter heart valves (THVs) remains a major challenge, as the gradual deterioration of biological valve leaflets due to calcification and thrombosis can lead to an early failure of the implanted device [6,7]. The durability of THVs can be attributed to several failure mechanisms such as leaflet thrombosis and infective endocarditis [8], paravalvular leaks [9,10], leaflet tears [11] and structural failure [12], which can result in increased patient morbidity and mortality [13].

Diagnostic imaging at follow-up represents the standard to evaluate the performance of THVs, but it is restricted to a morphological analysis rather than a functional assessment. Another imaging modality is transesophageal echocardiography, which is an invasive assessment of device function. Recent advances in sensor miniaturization and telemetry accelerated the development of remote telemonitoring systems enabled by an embedded sensor to monitor mechanical prosthetic heart valves using the electrical impedance across the valve [14]. Moreover, the attention towards computational modeling and simulations for testing biomedical devices, as opposed to in vitro or in vivo analyses, is growing considerably. This growth aims to improve not only the design phase but also the efficacy of the devices once implanted. In TAVI, the fluid–solid interaction (FSI) numerical technique was developed to quantify the structural and hemodynamic behavior of THVs using patient-specific models [15,16]. Structural parameters can be estimated from numerical simulations to quantify the performance of the implanted device, whereas hemodynamic quantification enables an estimation of critical TAVI-related parameters such the pressure gradient across the device. Fiber optic sensors offer advantages in biomedical applications due to their flexibility, small size and immunity to electromagnetic interference. Kuang et al. [17] integrated low-cost plastic optical fibers into smartphones to monitor human physiological signals, demonstrating the feasibility of measuring the heart rate and breathing rate. Savovic et al. [18] investigated the influence of bending on transmission in optical fibers, noting that the effects become significant when the bend radius decreases below approximately 0.04 m. Among the many optical sensors available, the photoplethysmography (PPG) sensors are capable of measuring blood volumetric changes in the subcutaneous vessels [19]. Thus, sensors can be potentially used to detect the changes in the flow field if properly positioned.

The central hypothesis of this study is that an appropriately configured system of optical fibers and PPG sensors can be adopted to quantify the structural and hemodynamic behavior of SAPIEN 3 Ultra (S3) THV, with the ultimate goal of assessing biological tissue leaflet degradation. We employed computational modeling to evaluate the feasibility of utilizing optical fiber and PPG sensors for monitoring device performance, based on predictions of flow and strain. This study demonstrated that computational modeling could facilitate the optimal design and positioning of wearable devices for the in vitro evaluation and monitoring of flow performance in THVs.

## 2. Materials and Methods

### 2.1. Model Geometry

**Figure 1:**
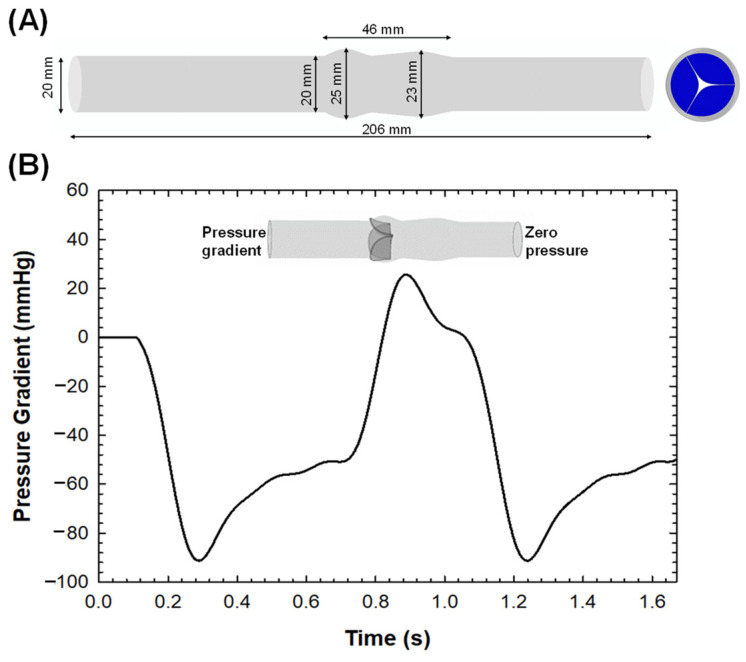
(**A**) Dimensions of CAD model of idealized vessel with cross-section of native valve and (**B**) FSI boundary condition of time-dependent pressure gradient applied to the inlet surface for simulating fluid motion.

The Rhinoceros computer-aided design (CAD) software (Rhinoceros v.7, McNeel and associates, Seattle, WA, USA) was employed to generate a CAD model of an idealized native aortic vessel. The model encompasses a 3D geometry of the tricuspid heart valve, comprising the cusps, the root and the sinuses, consistent with the morphological characteristics and dimensions of a normal heart. The idealized vessel had a valve annulus diameter of 20 mm and sinus diameter of 25 mm. Similarly, the shape of the aortic valve leaflets represented the morphological shape of a normal aortic root, as previously described [20]. To ensure a uniform flow regime, the proximal side of the left ventricle outflow tract and distal portion of the ascending aorta were extended by eight times the size of the vessel diameter. Figure 1A shows the dimensions of the CAD model of the idealized vessel.

To simulate a transcatheter heart valve implantation (TAVI) procedure, the 23 mm SAPIEN 3 Ultra (S3) device (Edwards Lifescience, Irvine, CA, USA) was considered. The S3 device is a balloon-expandable device composed of a stent frame, a tri-leaflet valve and a fabric skirt. The stent frame is made from cobalt–chromium material, the valve is a processed bovine pericardial tissue and the skirt is made from polyethylene terephthalate material. The CAD model of the S3 device was generated combining geometrical measurements collected from a high-resolution micro-computed tomography scanner with the reverse engineering of the metallic stent frame. The device valve leaflets were designed in agreement with CAD models described by Morganti et al. [21].

### 2.2. Simulation

The multiphysics simulation of TAVI was carried out using fully coupled two-way FSI analysis to estimate measurements commonly used for evaluating the bioprosthesis performance. First, the deployment of the S3 device on the ideal vessel was determined via finite-element analysis using Abaqus/Explicit software (v2021hf7, Dassault Systèmes, Johnston, RI, USA). Then, a two-way FSI analysis was developed to simulate the post-TAVI device performance using coupling Abaqus v2021hf7 (with a restart analysis) with an XFlow 2022 lattice Boltzmann fluid solver (Dassault Systemes, Simulia, USA). The lattice Boltzmann Method is a computational approach used in fluid dynamics simulations, where the fluid is represented by a collection of particles distributed on a discrete lattice grid. The particles move and interact according to simplified kinetic rules, which include the following: (a) collision, where particles’ velocities are adjusted towards equilibrium using a multi-relaxation time (MRT) technique [22] and (b) streaming, where particles move to neighboring grid points, using the bounce-back method near walls [23].

#### 2.2.1. TAVI Structural Simulation

A quasi-static finite-element analysis was carried out by ensuring that the ratio of the kinetic to internal energy remained below 10%. This was achieved through a mass scaling technique using a stable time increment of 1.0 × 10^−6^ every 100 iterations. In addition, a penalty contact algorithm with a friction coefficient of 0.1 was used to enable contact during simulations among valve leaflets. The stent frame was meshed with structured hexahedral elements, as reported in previous studies by our group [24,25]. Stainless steel with bilinear elasto-plastic material was employed to model the device metallic stent frame. Linear elastic material properties with a Young modulus of 4 MPa were used for the aortic wall and native valve leaflets. The aortic wall was assumed to be a shell part with quadrilateral elements (S4R) and a material thickness of 2 mm. For native valve leaflets, a 6-node linear triangular prism mesh (C3D6) was developed using extruding shell elements with four layers through-the-thickness. The latter was assumed to be 0.5 mm thick. After simulating the deployment of the stent frame, the skirt was modeled by closing the stent frame cells with patch surfaces. These patch surfaces were connected to the device frame using tie contact conditions. Thus, the valve leaflets of the S3 device were mapped on the deformed stent frame. The Ogden constitutive law with a 2nd order polynomial form was adopted to model the biomechanical response of the pericardial tissue of device valve leaflets using μ1 = 0.00096 MPa, α1 = −56, μ2 = 3.57 MPa, α2 = 1.87 and D1 = D2 = 0.027 MPa [26].

The TAVI simulation involved simulating the device crimping, followed by the deployment of the S3 in the native valve leaflets, ending with a device recoil induced by vessel elastic material properties. The crimping of S3 under frictionless contact conditions was carried out using a cylindrical surface gradually moved along the radial direction from the initial device diameter of 23 mm to the final diameter of the closed device. After crimping, an elastic recoil of S3 Ultra was allowed to decrease internal energy before simulating the valve deployment. For the sake of simplicity, the expansion of the S3 Ultra THV was performed by radially displacing a cylindrical surface representing the wall of the expanding balloon. Expansion was obtained by enlarging the balloon surface upon the device nominal diameter of 23 mm. As boundary conditions, the proximal and distal ends of the aortic wall were fixed in all directions of the vessel. A viscous pressure of 1.0 × 10^−6^ MPa was used on the inner surface of device valve leaflets.

#### 2.2.2. Post-TAVI Two-Way FSI Simulation

FSI analyses were modeled using the lattice Boltzmann finite-element approach in the 3D model of the idealized vessel for the analysis of the interaction between the fluid domain and solid parts (i.e., the aortic wall and native valve leaflets). Specifically, the FSI was performed to simulate not only the native valve kinematic behavior but also the post-TAVI device performance.

After the device deployment, a restart analysis was developed in Abaqus to simulate the cardiac beat of 0.8 s. This restart analysis allowed us to account for a structural simulation setting as well as the initial stress state at the end of the implanted device. To allow the exchange of data between the structural and fluid solver, a surface was defined to include the device valve leaflets and aortic wall shapes. This represents the FSI co-simulation interaction enabling the exchange of fluid forces and deformations among different solvers.

For the fluid setup, the deformed configuration of the implantation scenario was imported as a stereolithography (STL) format in the XFlow solver. The blood was treated as Newtonian and isothermal at a temperature of 37 °C, with a dynamic viscosity of 0.0037 Pa-s and a density of 1060 kg/m^3^. A convergence analysis was conducted to obtain the optimal temporal and spatial discretization, where the peak velocity and max pressure gradient metrics convergence were used to calculate the optimal lattice size and time step. Thus, particles were distributed in the fluid domain with a spatial resolution of 0.001 m and a temporal resolution of 4.97 × 10^−5^ s, since the convergence was obtained for a spatial and temporal resolution ratio of dx/dt = 20. For the FSI simulation of native valve leaflets, the flow simulation was discretized with ~1 M unity D3Q27 lattices (27 × 28 × 209), with a high number of degrees of freedom per discrete element of fourth-order spatial discretization. A slightly different discretization was determined for the post-TAVI FSI simulation with a D3Q27 spatial discretization of 65 × 66 × 518. As boundary conditions, the pressure gradient between the left ventricle and the aorta (Figure 1B) was applied to the inlet surface, while the outlet surface had a zero-pressure outflow condition. A no-slip boundary condition was applied to the vessel wall. Two cardiac cycles were simulated for each FSI analysis.

### 2.3. Structural and Flow Measurement

An ideal polymeric optical fiber was integrated along the vessel circumference to provide the structural deformation of the vessel. Optical fiber sensors, owing to their sensitivity to strain and temperature, are increasingly being employed in the biomedical field [27] due to their small size, light weight, wearability, flexibility and compatibility with biological tissues. The fiber was virtually positioned downstream the valve, along the circumferential direction of the vessel, as this region was the area with the highest deformation from the FSI simulation of the healthy scenario. The fiber was meshed with truss elements (T3D2), assuming a linear elastic material behavior with Young’s modulus of E = 3000 MPa and Poisson’s coefficient of ν = 0.49 [28]. Tie contact constraints were used to connect the fiber to the aortic vessel wall.

Once the post-TAVI FSI simulation was performed, blood pressure estimates were used to optimize the position of PPG sensors for evaluating device performance. Specifically, we placed two PPG sensors that were located proximally and distally to the bioprosthesis. A script to investigate the effect of the pressure gradient across the implanted device on pulse wave velocity (*PWV*) and pulse transit time (PTT) was developed using the mathematical language program MATLAB (R2021a, The MathWorks Inc., Natick, MA, USA). The script leverages principles from fluid dynamics, particularly the Bramwell–Hill equation, which provides the relationship between arterial distensibility and PWV to model the relationship between predicted pressure gradient, distance among sensors and *PWV*:

(1) $$PWV=\sqrt{\frac{\Delta P}{\rho }\frac{A}{\Delta A}}$$

where Δ*P* is the pressure gradient across the bioproshtesis, *A* is the mean area of the blood vessel just after the device, Δ*A* is the difference between the maximum and minimum vessel area just after the device during the cardiac cycle and ρ represents the blood density. Differently, the PTT was calculated as the ratio of the distance between the PPG sensors to the *PWV*.

A parametric analysis was performed to quantify the effect of different pressure gradients on PPG signal and distances among PPG sensors. The script considers a range of distances between the PPG sensors, from 10 mm to 70 mm, to simulate different scenarios that can be replicated in experimental settings. The pressure gradients ranged from 4 to 15 mmHg, corresponding to the physiological range of pressure gradients across the S3 device. Understanding how *PWV* and PTT vary within this range can provide insights into the function of the aortic valve, particularly in distinguishing between normal function and functional stenosis.

## 3. Results

**Figure 2:**
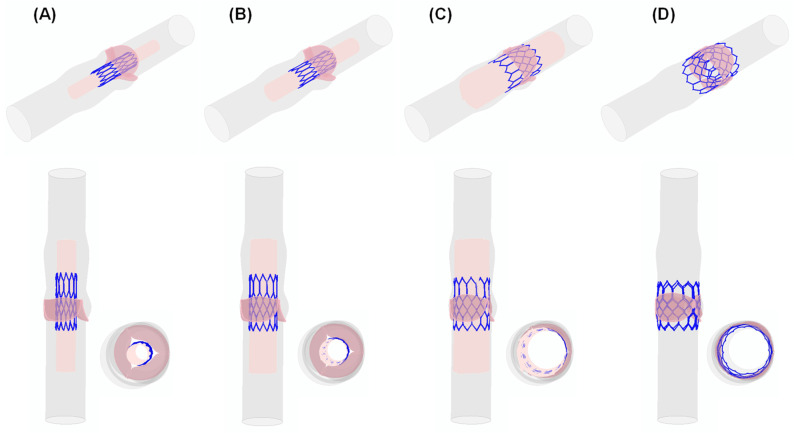
Different steps of TAVI simulation; the deployment from the crimped THV to (**A**–**D**) expanded SAPIEN 3 Ultra: (**A**) Initial positioning of the crimped THV within the catheter. (**B**) Early stages of deployment as the catheter begins to release the THV. (**C**) Mid-deployment phase where the THV continues to expand further, making contact with the walls of the aorta. (**D**) Full deployment of the THV where the valve is completely expanded and positioned within the aorta.

Several steps of the device deployment are shown for both the axial and sagittal views of the idealized aortic vessel (Figure 2). Firstly, the transcatheter heart valve (THV) is in a crimped state prior to deployment within the aorta (Figure 2A). In the second step (Figure 2B), the valve begins to expand slightly as the crimping pressure is released. Subsequently, the structural frame of the valve starts to assume the intended shape (Figure 2C) until the valve frame fully engages with the aortic walls, ensuring proper anchoring and alignment (Figure 2D). Thus, the device valve leaflets were mapped into the metallic stent frame to simulate the cardiac beat.

**Figure 3:**
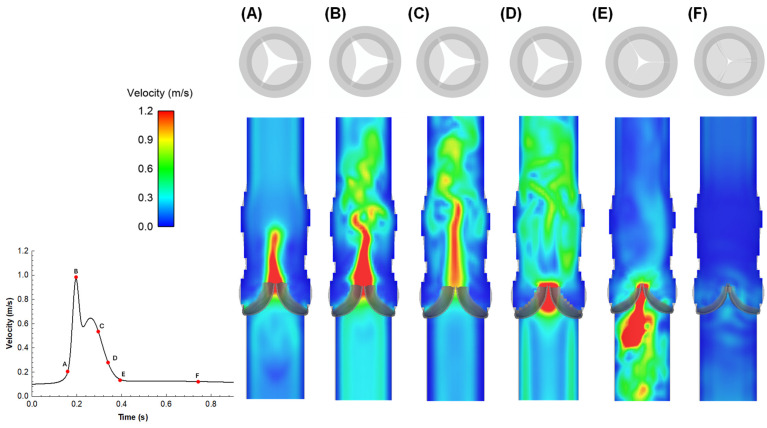
FSI models using coupled LBM-FE: flow velocity at representative time points during native heart valve cardiac cycle (**A**–**F**). (**A**) Blood flow acceleration; (**B**) peak systole; (**C**,**D**) flow deceleration; (**E**) early diastole just prior the valve closure; (**F**) long diastole.

**Figure 4:**
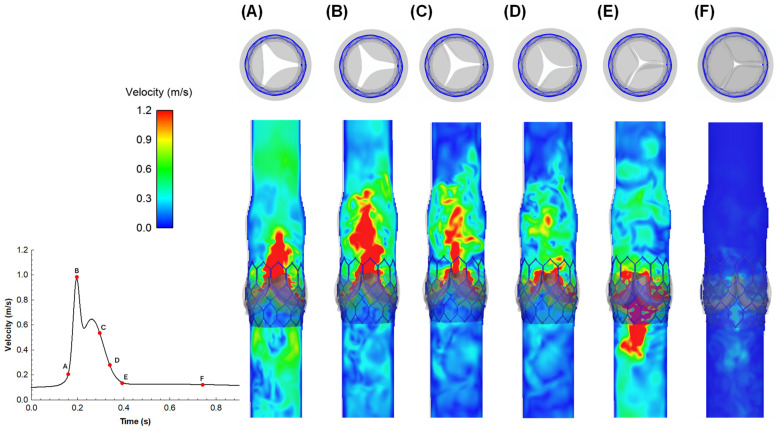
FSI models using coupled LBM-FE: flow velocity field at representative time points (**A**–**F**), post-TAVI procedure. (**A**) Fluid acceleration, (**B**) systolic peak; (**C**,**D**) deceleration; (**E**) early diastole and (**F**) long diastole.

The flow velocity field predicted using the multiphysics FSI simulation is presented at different time steps of the cardiac cycle for the ideal vessel with the native valve (Figure 3) and the bioprosthesis (Figure 4).

For the pre-TAVI simulation, at the beginning of the systole phase (Figure 3A), the flow jet accelerates (Figure 3B) to show the maxima at systolic peak. The flow field is characterized by a symmetric central jet with a maximum of approximately 1.2 m/s (Figure 3C). During deceleration, a backward flow due to pressure decrease resulted in leaflet partial closure and flow acceleration showing a physiologically normal regurgitation (Figure 3D). As the fluid is forced to change direction or stop abruptly, a pressure drop is generated across the native aortic valve. During early diastole (Figure 3E), the velocity decreases, showing small flow magnitudes above the native aortic valve until flow velocity drops approximately to zero (Figure 3F). For the post-TAVI simulation, the overall flow pattern was similar to that of the native aortic valve, indicating that the bioprosthesis serves to restore the physiological hemodynamic. Specifically, at the early systole phase (Figure 4A), the flow velocity field shows high-velocity regions near the central area of the valve, indicating the beginning of turbulent flow patterns as blood starts to be ejected through the newly deployed THV. At the mid-systole phase (Figure 4B), the velocity field begins to stabilize, with high-velocity regions more evenly distributed around the valve. In particular, the high flow values occurring on the valve leaflet surfaces are likely caused by numerical instabilities induced by the numerical method. In the late systole phase (Figure 4C), the flow velocity exhibits a more uniform distribution, indicating that the valve leaflets are fully opened and the flow is becoming more streamlined. This trend continues until the end of the systole phase (Figure 4D), where a further stabilization of the flow field is observed. When the early diastole phase occurs (Figure 4E), the valve leaflets begin to close and the flow velocity field shows a remarkable reduction in velocity. At the late diastole (Figure 4F), the low-velocity regions near the closed valve indicate the valve capability to prevent backflow.

**Figure 5:**
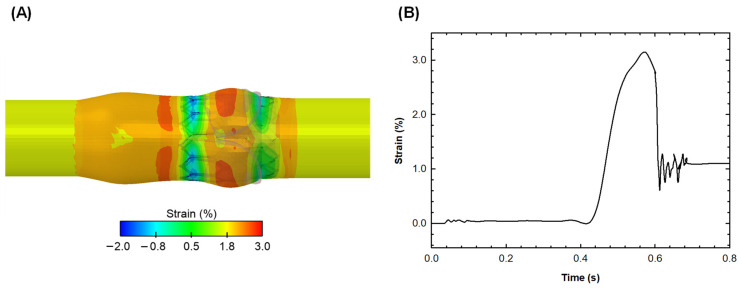
Contour plot of circumferential engineering strain felt by the vessel (**A**) and strain (%) as a function of time of the polymer optical fiber (**B**).

After the TAVI procedure, the distribution of the engineering strain in the circumferential direction was analyzed to determine the vessel region at its highest deformation and thus the optimal positioning for the optical fiber sensor (Figure 5A).

The local maxima of the circumferential strain occurred near the sinuses and sino-tubular junction at peak systole, in the contact area of the aortic root with the optical fiber downstream of the valve, as well as in the contact area between the aortic root and native valve leaflets. Figure 5B shows the profile of the circumferential strain along the optical fiber length as a function of time. At peak systole, a peak circumferential strain of 3% was observed when the optical fiber was placed along the sino-tubular junction.

**Figure 6:**
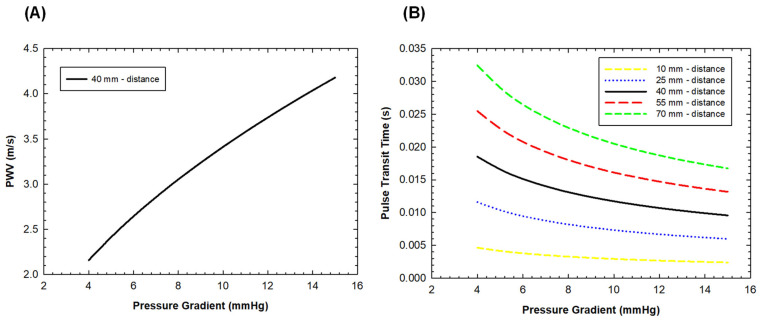
Estimations of PWV for the optimal distance between the PPG sensor (40 mm) (**A**) and estimations of PTT for different distances between PPG sensors (**B**) as a function of the transvalvular pressure gradient.

Figure 6 displays the estimations of PPT changes for different distances among PPG sensors and PWV with the pressure gradient across the bioprosthesis.

It can be observed that the pressure gradient across the bioprosthesis significantly influences the PWV estimations. Specifically, as the pressure gradient increases, the *PWV* estimations also increase. This relationship is explained by the Bramwell–Hill equation (Equation (1)), which shows that *PWV* is proportional to the square root of the pressure gradient. Conversely, the PTT estimates change with the distance between the PPG sensors. As the distance between the proximal and distal PPG sensors placed around the implanted device increases, the PTT estimates also increase.

## 4. Discussion

In this study, a multiphysics analysis within an ideal vessel featuring a balloon-expandable THV was carried out to estimate key structural and hemodynamic parameters, encompassing flow velocity, blood pressure and vessel wall deformation. These parameters serve as pivotal metrics for evaluating the performance of bioprosthesis and are instrumental in assessing the appropriate sizes of PPG and fiber optic sensors. Simulations revealed the optimal positioning of the polymeric fiber in the idealized vessel, maximizing the post-TAVI deformation of the aortic wall. Furthermore, our examination of the distances between the PPG sensors demonstrated the effectiveness of positioning sensors both upstream and downstream of the bioprosthesis for measuring the pressure gradient across the valve. While our study remains confined to a numerical investigation within an idealized vessel geometry, the proposed in silico approach facilitates the design of a sensor-embedded phantom model for the in vitro evaluation and monitoring of valve-related performance. This holds particular significance for THVs with an anticipated lifespan of nearly five years. Thus, the present findings may assist in the early identification of valve degeneration and, consequently, guide prophylactic intervention.

As THVs are increasingly implanted in young patients, the expectation for device durability surpasses the initial patient population involved in clinical trials assessing TAVI safety and efficacy. There exists a growing concern regarding the gradual deterioration of biological valve leaflets due to calcification and thrombosis, with reported incidences as high as 9.5% after three years post-implantation [29]. Consequently, there is an urgent need for the development of innovative approaches to monitor the degeneration of THVs. In this context, Bailoor et al. [30] demonstrated the feasibility of monitoring bioprosthesis hemodynamic performance using two pressure sensors positioned within the stent frame, at mid-height and at the distal portion of the device. They initially employed a computational model to predict the pressure field in an ideal vessel model and then adopted machine learning techniques to predict leaflet mobility and classify healthy versus diseased valves. Our study aims to assess valve function using PPG sensors and analytical considerations to estimate the pressure gradient across the valve. Unlike previous methods, we integrate PPG sensors to gain deeper insights into how *PWV* and PTT vary within the provided pressure gradient range, offering insights into aortic valve function and ultimately distinguishing normal from abnormal function. Furthermore, our investigation at different relative distances among PPG sensors highlighted a critical aspect for clinical application. While our aim was to position two sensors proximally and distally to the bioprosthesis to maximize the pressure gradient generated by leaflet mobility, inherent anatomical constraints may present challenges in achieving sensor placement in proximal positions. Through an analytical parametric study of the optimal distance between PPG sensors, we estimated that the optimal distance could be 70 mm, but this distance may cover nearly the entire length of the aorta and left ventricular outflow tract. However, reducing the distance between PPG sensors to 40 mm could strike a better balance between measurements and anatomical constraints.

After conducting multiphysics simulations, we found that the predicted circumferential strain (i.e., nearly 3%) aligns well with documented distensibility patterns of the aorta in elderly individuals [31]. Strain estimates obtained through FSI simulations are consistent with the full range of measurements achievable with optical fiber polymer or Bragg sensors. It is widely recognized that the biomechanical properties of the aortic wall tissue begin to decline after the age of 50. The aortic wall tends to stiffen due to changes in the collagen and elastin content of the extracellular matrix, leading to an increase in Young’s modulus of the vessel wall. By analyzing dynamic computed-tomography data, we demonstrated that the circumferential strain typically falls within the range of 2–6% in patients with aneurysmal aortas. Similarly, Satriano et al. [32] observed the peak principal strain in the range of 3–8% for TAVI patients with a mean age of 81 ± 6 years. They also established a correlation between the severity of aortic valve stenosis and vessel wall strain, where low valve orifice areas corresponded to low strain values. It is noteworthy that pronounced strain values were observed in the sinuses of Valsalva in the present investigation. However, this location may not be ideal for estimating vessel deformation due to the natural curvature of the sinuses, which could introduce bending effects influencing the measurement. Therefore, the optimal region for measuring vessel wall deformation appears to be 1 cm distal to the sino-tubular junction, where strains remain high.

One limitation of our computational investigation pertains to the use of homogeneous isotropic material properties for both the aortic vessel and native valve leaflets. Though aortic root is anisotropic, Bosi et al. [33] validated the use of linear elastic material properties for the aortic root against imaging data of TAVI patients. An Ogden’s constitutive model for the pericardial tissue of the device valve leaflets was adopted, as this yielded a more stable solution during the FSI simulation compared to fiber-reinforced constitutive modeling. The natural curvature of the human aorta, as opposed to the straight ideal model, may influence the strain field in circumferential directions. Exploring the sensitivities of the proposed sensors could help pinpoint the localization of aortic wall regions with critical stress, thereby enhancing model development. The concept outlined in this study aims to demonstrate how in silico multiphysics simulations can be utilized to optimize sensor embedment in an in vitro aortic model. Furthermore, this approach holds promise for improving the evaluation of THVs, particularly in identifying biological leaflet deterioration and developing strategies for prompt valve repair.

## 5. Conclusions

This study provides significant insights into the evaluation of the optimal sensor positioning for valve monitoring and detecting biological leaflet degradation following TAVI. Specifically, the multiphysics simulation was developed to quantify structural and fluid-dynamic parameters linked to the performance of THVs. The computational approach enabled the designing of sensors for quantifying the vessel wall strain and pressure gradient across the valve leaflets, with the ultimate goal of developing a low-cost monitoring system to detect valve deterioration.

## Data Availability

Dataset available on request from the authors.
